# Exposure to air pollution and ovarian reserve parameters

**DOI:** 10.1038/s41598-023-50753-6

**Published:** 2024-01-03

**Authors:** Katarzyna Wieczorek, Dorota Szczęsna, Michał Radwan, Paweł Radwan, Kinga Polańska, Anna Kilanowicz, Joanna Jurewicz

**Affiliations:** 1 Department of Chemical Safety, Nofer Institute of Occupational Medicine, 8 Teresy St; 91-348, Łódź, Poland; 2 Department of Gynecology and Reproduction, “Gameta” Hospital, 34/36 Rudzka St; 95-030, Rzgów, Poland; 3 Faculty of Health Sciences, Mazovian State University in Płock, 2 Dabrowskiego Sq; 09-402, Plock, Poland; 4 Department of Gynecology and Reproduction, “Gameta” Health Centre, 7 Cybernetyki St; 02-677, Warsaw, Poland; 5 Department of Gynecology and Reproduction, “Gameta” Clinic, Kielce-Regional Science –Technology Centre, 45 Podzamcze St; 26-060, Chęciny, Poland; 6 Department of Paediatrics and Allergy, Copernicus Memorial Hospital, Medical University of Lodz, Piłsudskiego 71; 90-329, Lodz, Poland; 7 Department of Toxicology, Medical University of Lodz, Muszynskiego 1; 90-151, Łódź, Poland

**Keywords:** Public health, Endocrine reproductive disorders

## Abstract

Exposure to air pollution is associated with many different health effects, especially cardiovascular and respiratory diseases. Additionally, highly significant links between exposure to air pollution and fertility, particularly male fertility was observed, however the studies regarding exposure to selected air pollutants and female fertility assessed by ovarian reserve are rare. Hence, the main aim of the study was to analyze relationship between exposure to ambient air pollution and ovarian reserve parameters among Polish women. The study population consisted of 511 women, who attended to infertility clinic because of diagnostic purposes. Participants filled in the questionnaire about social-demographic, lifestyle and health factors. Infertility specialists assessed ovarian parameters such as: antral follicle count (AFC) and concentration of hormones: Anti-Müllerian hormone (AMH), follicle stimulating hormone (FSH) and estradiol (E2). The air pollutants level (sulfur dioxide, nitrogen dioxide, carbon monoxide, ozone, particulate matters) were obtained via National Environmental Protection Inspectorate database. Significant negative association between PM_2,5_ and AMH (p = 0.032), as well as AFC (p = 0.044) was observed. Moreover, SO_2_ concentrations decrease AFC (p = 0.038). The results also suggest that PM_10_, PM_2.5_, SO_2_ exposure on antral follicle count may be more pronounced among women with a female factor infertility diagnosis. Additionally, exposure to PM_2.5_ and NOx on AFC and AMH was stronger among older women (> 35 years of age). To conclude, the present study found that air pollution could lead to decrease in follicle antral count and Anti-Müllerian hormone level, especially exposure to PM_2,5_ and SO_2_ thus the evidence suggest negative impact to ovarian reserve.

## Introduction

Currently ambient air pollution caused a serious environmental problem in many countries. The sources can be anthropogenic or natural. The pollutants of anthropogenic origin include: automotive industry, metallurgic industry and energy industry, whereas the natural sources include: volcanic eruptions, fires, biochemical processes, flash of lightening etc.^1^. Most of the studies regarding air pollution exposure are focused on monitoring of the air parameters such as: sulfur dioxide (SO_2_), nitrogen dioxide (NO_2_), carbon monoxide (CO), ozone (O_3_), particulate matters (PM_2,5_ and PM_10_). A components of particulate matters (PM) can be organic (e.g. dioxins, benzene, polycyclic aromatic hydrocarbons) or inorganic (e.g. heavy metals, chlorides, carbon) origins^2^. According to the new report by European Environment Agency (Air quality in Europe 2022 report) in 2020 96% of the urban population in European Union was exposed to PM levels exceeded guidelines by the World Health Organization^3^. In Poland air pollution caused a huge problem, according to the World Bank Group 36 of the 50 most polluted cities in the European Union are located in Poland. The particulate matters (PM_2,5_) are mostly responsible for air pollution in these cities^4^.

Air pollution exposure can be associated with many different health effects, especially: respiratory or cardiovascular system diseases^5–7^. Increasing number of studies and reviews suggest a negative impact of air pollution on human reproduction including male and female fertility. A lower X:Y sperm chromosome ratio among men and abnormal morphology (51,78%) was observed after air pollution exposure^7^. Additionally, air pollution exposure negatively affects: DNA fragmentation^8,9^, sperm aneuploidy^10,11^, sperm concentration and sperm motility^8,12–18^. In comparison to male populations, female exposure to ambient air pollution and reproductive outcomes are not frequently studied, especially female fertility is rarely assessed. In the review performed by Conforti et al.^19^, the significant linkage between air pollution and female reproductive outcomes such as: higher miscarriages rate^20,21^, risk of stillbirth^22^ was observed. Additionally, Zeng et al.^23^ reported that, chronic exposure to air pollution can lead to reduced odds of IVF pregnancy outcomes (biochemical and clinical pregnancy). The studies assessing the female fertility are scare. The first study which evaluate the environmental exposure to air pollution (PM_2,5_) and one of the ovarian reserve parameters, antral follicle count (AFC) was performed by Gaskins et al.^24^. The study shows that exposure to particulate matters (PM_2,5_) can lead to decrease ovarian reserve. Other studies examined the exposure to particulate matter (PM_10_ and PM_2,5_) found the negative association with Anti-Müllerian hormone^25^ and decrease in AMH ratio, defined as an observed-to-expected AMH based on age^26^. In the study performed by Feng et al. long–term (between January 2013 and December 2019) exposure to SO_2_ was associated with lower antral follicle count^27^. In animals the studies also confirm that air pollution exposure may decrease fertility. The studies on mice suggest that, exposure to fraction PM_2,5_ can reduce the level of Anti-Müllerian hormone (AMH)^28^. Other researchers reported that, exposure to PM_2,5_ can affects antral follicle count, pregnancy loss and birth weight^29^.

In comparison to previous research this study is the first which describe all parameters of ovarian reserve (AFC, FSH, E2, and AMH) and different air pollutants (SO_2_, NO_x_, CO, O_3_, PM_2,5_ and PM_10_) exposure. Thus this study aims to evaluate the relationship between air pollution exposure and ovarian reserve.

## Materials and methods

### Study population

The study group include 511 women in reproductive age (25–39 years of age) recruited from fertility clinic. Only menstruating women with ovulatory cycles without co-existing chronic diseases (e.g., fragile X syndrome, adrenocortical insufficiency, abnormal karyotype) were included. Women with: three spontaneous miscarriages, more than three in vitro fertilization procedures, chemotherapy or radiotherapy of pelvis, premature ovarian failure, previous surgical treatment of the ovaries, polycystic ovary syndrome, cyst in the ovaries with endometrium, hyperprolactinemia, hypogonadotropic and hypogonadism were excluded. 700 woman fulfill the inclusion criteria and were suitable for the research, however only 511 (73%) gave the approval to participate in the research.

All the participants filled the questionnaire about socio-demographic characteristics, lifestyle factors (e.g. smoking, alcohol drinking, physical activity), health conditions (e.g. co-existing diseases), and occupational exposures.

This study was approved by the Nofer Institute of Occupational Medicine Bioethical Committee Board (ethics approval number: Resolution 23/2016). All experiments were performed in accordance with relevant guidelines and regulations. Informed consent was obtained from all subjects.

### Analysis of ovarian reserve parameters

In the study ovarian reserve parameters were assessed such by antral follicular count (AFC), and reproductive hormones level (AMH, FSH, and estradiol).

The count of the antral follicle was assessed by ultrasonography (USG) according to Broekmans et al. criteria^30^. To assessment considered antral follicle with dimension measurement extent from 2 to 10 mm. All procedure was performed by trained gynecologist. The antral follicle count was treated for the analysis as the sum of antral follicle in both ovaries.

The blood samples were collected in early follicular phase cycle of spontaneous menstruation (2–4 day of cycle). The concentrations of the following hormones were assessed in serum: AMH (Anti-Müllerian hormone), FSH (follicle stimulating hormone) and estradiol (E2). The blood samples were centrifuged and a serum collected to polypropylene test tubes and stored in − 80 °C to time of analysis.

An enzyme linked immunosorbent method was used to analyzed the level of AMH using Gen-II ELISA kits (Beckman Coulter, Inc., USA) based on manufacturer instruction. The chemiluminescence method was used to measure levels of FSH and estradiol from ITROS ECi Immunodiagnostic System with MicroWell technology using commercially available VITROS Reagent Packs and the VITROS Calibrators which was used based on manufacturer instruction (Ortho-Clinical Diagnostics Johnson & Johnson, UK).

### Air pollution data and exposure assessment

The air pollutants concentrations were obtain the National Environmental Protection Inspectorate (https://powietrze.gios.gov.pl/pjp/current). Daily levels (24-h average) of particulate matter < 10 μm in aerodynamic diameter (PM_2.5_), particulate matter > 10 μm in aerodynamic diameter (PM_10_), and sulfur dioxide (SO_2_) (reported as μg/m3) were collected. Additionally, the carbon monoxide CO (reported as μg/m3) and ozone (reported as μg/m^3^) measured in maximum 8-h average and nitrogen dioxide NOx (reported as μg/m^3^) -maximum 1-h average. Also meteorological factors such as temperature, atmospheric pressure, humidity, wind direction were collected.

For each pollutant, the average value for the 6 months which address the period of developmental stage from primary follicle to antral follicle, nearest monitoring sites was assigned to the women according to their ZIP code of residence.

### Statistical methods

Descriptive statistics were calculated for subjects grouped by demographic characteristics, along with the layout of examined air pollutants, and AMH, E2 and FSH levels and AFC. Multiple least squares linear regression models were used to quantify the relationship of air pollution exposure (explanatory variables) with AFC and the concentrations of AMH, E2 and FSH as dependent variables. Multivariate regression models was used to explore an association between air pollutants levels, reproductive hormone concentrations and AFC. Two models were constructed. The first model was adjusted for following variables: BMI (kg/m^2^), age (years), smoking (no/yes), initial infertility diagnosis (male factor; female factor; unexplained) and the second model for age, BMI, smoking, infertility diagnosis, duration of infertility (1–3 years; 3–5 years; > 5 years); alcohol consumption (none or < 1 drink/week; 1–3 drinks /week; everyday). The covariates in the model were included on biological and statistical consideration. The air pollutants in the model were treated as the categorical variable (first to 25th percentile value, second-greater than the 25th percentile value to the median, third greater than the median to 75th percentile value, while the fourth group consisted of values greater than the 75th percentile or continuous variable. Effect modification of the association between exposure to air pollution and ovarian reserve parameters by age, BMI, current smoking, infertility diagnosis, duration of infertility, all well-known predictors of ovarian reserve, by adding a cross-product term to the final multivariate model was tested. R statistical software was used for the analysis (version 4.2.2).

### Ethics approval and consent to participate

The Bioethical Committee in Lodz, Poland, approved the study (Resolution no 23/2016). All participants obtained and signed written informed consents prior to enrollment.

## Results

### Participants’ characteristics

Characteristics of the study population were described in Table 1. The majority of participants had higher (75.34%, n = 385) or secondary (21.14%, n = 108) education, the mean age was 33.30 ± 3.69 years and mean BMI (body mass index) 23.18 ± 3.80 kg/m^2^. The women were mostly nonsmokers (92.17%, n = 471), and 55% (281) of study subjects announced that they do not drink alcohol or drink less than 1 drink per week whereas 224 (44%) declare drinking 1–3 drinks per week. The initial diagnosis of infertility during recruitment was: male factor (37.8%, n = 193), idiopathic infertility (31.1%, n = 159), endometriosis (13.7%, n = 70), ovarian factor (4.7%, n = 24) and tubal factor (10.2%, n = 52). The duration of couple’s infertility declared by study participant were: over 5 years (35.23%, n = 180), 3–5 years (29.55%, n = 151), 2–3 years (27.59%, n = 141) and 1–2 years (7.63%, n = 39).

**Table 1:** Characteristics of the study population N = 511.

Variables		N (%)	Mean ± SD
Education	Vocational	18 (3.52)	
	Secondary	108 (21.14)	
	Higher	385 (75.34)	
Age [years]	24–30	121 (23.68)	33.30 ± 3.69
	31–39	390 (76.32)	
Body mass index $\left[\frac{\mathbf{k}\mathbf{g}}{{\mathbf{m}}^{2}}\right]$	< 18.5	29 (5.68)	23.18 ± 3.80
	18.5–24.9	301 (58.90)	
	25–29.9	154 (30.14)	
	30–40	27 (5.28)	
Current smoking	No	471 (92.17)	
	Yes	40 (7.83)	
Alcohol use	None or < 1 drink/week	281 (55.0)	
	1–3 drinks/week	224 (44.0)	
	Everyday	6 (1)	
Initial infertility diagnosis	Male factor	193 (37.8)	
	Idiopathic	159 (31.1)	
	Endometriosis	70 (13.7)	
	Ovarian factor	24 (4.7)	
	Tubal factor	52 (10.2)	
	Missing data	13 (2.5)	
Duration of couple’s infertility [years]	1–2	39 (7.63)	
	2–3	141 (27.59)	
	3–5	151 (29.55)	
	> 5	180 (35.23)	

### Ovarian reserve parameters

Table 2 presents ovarian reserve parameters among study population. For AFC arithmetic mean (± SD) was 12.73 ± 8.94. Reproductive hormones levels were: 1.17 ± 1.46 (ng/ml) for Anti-Müllerian Hormone, 6.38 ± 2.18 (IU/l) for follicle stimulating hormone and 93.74 ± 16.63 (pg/ml) for estradiol. According to the guidelines the AFC, FSH was in normal range (range for AFC: more than 4 antral follicles in ovary and range for FSH: lower than 15 IU/l). Additionally, estradiol for follicular phase of cycle was in normal range (range for E2: 12,5–166 pg/ml), the same as AMH (range for AMH: lower than 1 ng/ml).

**Table 2:** Ovarian reserve parameters among study population.

Ovarian reserve parameters	A Mean ± SD	G Mean ± SD	Min	Q25	Median	Q75	Q95	Max
AFC (n)	12.73 ± 8.94	12.25 ± 1.73	1	8	11	20	30	40
AMH (ng/ml)	1.17 ± 1.46	1.21 ± 1.4	0.02	0.9	1.3	2.9	9.36	18
E2 (pg/ml)	93.74 ± 16.63	91.33 ± 12.89	75	83	95	120	180	200
FSH (IU/l)	6.38 ± 2.18	6.00 ± 1.43	0.9	4.86	6.14	7.51	10.48	13.5

*A Mean* arithmetic mean, *G Mean* geometric mean, *SD* standard deviation, *Min* minimal value, *Max* maximum value, *Q25* 25 percentile, *Q75* 75 percentile, *Q95* 95 percentile, *AMH* Anti-Müllerian hormone, *AFC* antral follicle count, *FSH* follicle-stimulating hormone, *E2* estradiol.

### Ambient air pollution levels

The levels of ambient air pollution are presented in the Table 3. A geometric means were: 44.80 ± 18.21 µg/m^3^ (range: 12.31–88.12 µg/m^3^) for ozone, 38.98 ± 31.14 µg/m^3^ (range: 8.21–117.56 µg/m^3^) for PM_10_, 31.22 ± 21.12 µg/m^3^ (range: 9.21–90.12 µg/m^3^) for PM_2,5_, 41.56 ± 9.82 µg/m^3^ (range: 11.32–170.66 µg/m^3^) for SO_2_, 35.70 ± 24.60 µg/m^3^ (range: 1.45–174.89 µg/m^3^) for NO_x_ and 0.48 ± 0.32 µg/m^3^ (range: 0.11–1.98 µg/m^3^) for CO. According to Regulation of the Minister of the Environment of August 24, 2012 on the levels of certain substances in the air, almost all levels of air pollution^31^ in the study were fulfilled (Ozone range: 120 µg/m^3^, PM_10_ range: 50 µg/m^3^, SO_2_ range: 125 µg/m^3^, NO_x_ range: 30 µg/m^3^, CO range: 10,000 µg/m^3^). Only level of PM_2,5_ was run over (range: 20 µg/m^3^).

**Table 3:** Ambient air pollution level.

Air pollutants	Statistic						
	G Mean ± SD	Min	Q25	Median	Q75	Q95	Max
Ozone (µg/m^3^)	44.80 ± 18.21	12.31	37.11	43.11	68.11	75.12	88.12
PM_10_ (µg/m^3^)	38.98 ± 31.14	8.21	17.94	37.12	52.17	95.77	117.56
PM_2.5_ (µg/m^3^)	31.22 ± 21.12	9.21	17.34	27.87	64.17	71.12	90.12
SO_2_ (µg/m^3^)	41.56 ± 9.82	11.32	22.16	41.50	45.78	78.12	170.66
NOx (µg/m^3^)	35.70 ± 24.60	1.45	27.13	30.11	28.30	81.87	174.89
CO (µg/m^3^)	0.48 ± 0.32	0.11	0.24	0.43	0.84	1.20	1.98

*G Mean* geometric mean, *Q25* 25 quartile, *Q75* 75 quartile, *Q95* 95 quartile.

### Parameters of ovarian reserve and exposure to air pollution

The Table 4 presented relationship between air pollution levels and parameters of ovarian reserve. The statistical model was adjusted for: age, BMI, smoking and infertility diagnosis. A significant associations between parameters of ovarian reserve and air pollution was observed in 4^th^ quartile of exposure to PM_2,5_ for antral follicle count (AFC) (p = 0.044) and Anti-Müllerian Hormone (AMH) (p = 0.032) compared to the first quartile, when the exposure was treated as the categorical variable. Additionally, statistically significant relationship was found between SO_2_ concentration and antral follicle count (AFC) (p = 0.038). No association was found between estradiol (E2) and follicle stimulating hormone (FSH) and any examined air pollutants (PM_2.5_, PM_10_, SO_2_, CO, NOx, ozone) in quartiles of exposure. When the air pollution exposure was treated in the model as a continuous variable no association were found between any air pollutants and any examined ovarian reserve parameters (Table 4). When the model was adjusted additionally for duration of infertility and alcohol consumption only exposure to PM_2,5_ in 4th quartile was related to decrease in antral follicle count number (p = 0.045) and AMH concentration (p = 0.048) (Table 4). Other air pollutants (SO_2_, PM_10,_ ozone, NOx, CO) were not significantly associated with ovarian reserve parameters.

**Table 4:** The association between air pollution levels and parameters of ovarian reserve- multivariate analysis.

Categories		AFC			AMH			FSH			E2		
		Coef	95% Cl	p	Coef	95% Cl	p	Coef	95% Cl	p	Coef	95% Cl	p
Ozone $\left(\frac{\mu g}{{m}^{3}}\right)$	Cont^a^	− 0.04	− 0.134; 0.055	0.409	− 0.041	− 0.114; 0.033	0.277	− 0.008	− 0.047; 0.031	0.677	− 0.08	− 0.273; 0.113	0.409
	Cont^b^	− 0.03	− 0.122; 0.067	0.520	− 0.028	− 0.117; 0.026	0.221	− 0.005	− 0.012; 0.044	0.786	− 0.03	− 0.265; 0.251	0.489
	Q2^a^	− 0.061	− 0.148; 0.026	0.168	− 0.038	− 0.106; 0.03	0.278	− 0.004	− 0.04; 0.032	0.826	− 0.012	− 0.203; 0.18	0.902
	Q2^b^	− 0.061	− 0.168; 0.031	0.198	− 0.042	− 0.123; 0.07	0.310	− 0.002	− 0.02; 0.078	0.786	− 0.014	− 0.278; 0.21	0.978
	Q3^a^	− 0.016	− 0.11; 0.077	0.732	0.04	− 0.035; 0.115	0.296	− 0.011	− 0.049; 0.028	0.586	− 0.007	− 0.157; 0.144	0.926
	Q 3^b^	− 0.014	− 0.09; 0.056	0.659	0.05	− 0.043; 0.114	0.278	− 0.014	− 0.032; 0.097	0.760	− 0.005	− 0.144; 0.131	0.879
	Q4^a^	− 0.052	− 0.132; 0.028	0.204	− 0.018	− 0.08; 0.045	0.584	− 0.011	− 0.044; 0.022	0.505	0.118	− 0.07; 0.309	0.218
	Q4^b^	− 0.044	− 0.178; 0.032	0.219	− 0.011	− 0.07; 0.052	0.423	− 0.017	− 0.078; 0.011	0.35	0.324	− 0.05; 0.327	0.233
PM_10_ $\left(\frac{\mu g}{{m}^{3}}\right)$	Cont^a^	− 0.012	− 0.07; 0.045	0.674	0.028	− 0.018; 0.074	0.235	0.015	− 0.008; 0.039	0.205	− 0.15	− 0.327; 0.027	0.096
	Cont^b^	− 0.017	− 0.05; 0.078	0.763	0.031	− 0.015; 0.065	0.267	0.017	− 0.012; 0.067	0.123	− 0.09	− 0.217; 0.013	0.078
	Q2^a^	0.006	− 0.073; 0.086	0.873	0.035	− 0.029; 0.099	0.279	− 0.01	− 0.043; 0.023	0.566	0.021	− 0.157; 0.199	0.818
	Q2^b^	0.007	− 0.097; 0.076	0.756	0.067	− 0.057; 0.127	0.329	− 0.03	− 0.048; 0.035	0.673	0.033	− 0.165; 0.145	0.852
	Q3^a^	− 0.027	− 0.109; 0.055	0.519	− 0.035	− 0.099; 0.029	0.288	0.033	− 0.001; 0.066	0.054	0.06	− 0.138; 0.259	0.541
	Q3^b^	− 0.032	− 0.115; 0.047	0.556	− 0.045	− 0.102; 0.032	0.167	0.056	− 0.011; 0.064	0.078	0.04	− 0.155; 0.321	0.589
	Q4^a^	− 0.033	− 0.079; 0.013	0.164	− 0.011	− 0.048; 0.025	0.544	− 0.002	− 0.022; 0.017	0.822	0.044	− 0.131; 0.22	0.615
	Q4^b^	− 0.051	− 0.082; 0.023	0.178	− 0.010	− 0.056; 0.039	0.487	− 0.005	− 0.012; 0.011	0.798	0.050	− 0.178; 0.34	0.656
PM_2.5_ $\left(\frac{\mu g}{{m}^{3}}\right)$	Cont^a^	− 0.006	− 0.078; 0.066	0.869	0.059	0.003; 0.116	0.241	− 0.025	− 0.055; 0.005	0.101	− 0.106	− 0.28; 0.067	0.223
	Cont^b^	− 0.010	− 0.089; 0.078	0.658	0.065	0.012; 0.185	0.378	− 0.022	− 0.021; 0.010	0.112	− 0.178	− 0.11; 0.078	0.356
	Q2^a^	− 0.024	− 0.062; 0.013	0.204	− 0.025	− 0.055; 0.004	0.092	0.004	− 0.012; 0.02	0.616	− 0.019	− 0.196; 0.16	0.838
	Q2^b^	− 0.028	− 0.069; 0.018	0.211	− 0.032	− 0.059; 0.002	0.078	0.004	− 0.011; 0.032	0.678	− 0.023	− 0.210; 0.22	0.876
	Q3^a^	− 0.030	− 0.18; 0.130	0.750	− 0.017	− 0.047; 0.013	0.273	− 0.015	− 0.053; 0.024	0.449	0.039	− 0.126; 0.205	0.642
	Q3^b^	− 0.028	− 0.167; 0.126	0.457	− 0.009	− 0.078; 0.041	0.398	− 0.017	− 0.021; 0.028	0.452	0.042	− 0.156; 0.236	0.789
	Q4^a^	− 0.040	− 0.28; − 0.010	**0.044**	− 0.009	− 0.014; − 0.002	**0.032**	0.004	− 0.012; 0.019	0.659	0.104	− 0.07; 0.278	0.238
	Q4^b^	− 0.030	− 0.31; − 0.013	**0.045**	− 0.011	− 0.013; − 0.003	**0.048**	0.005	− 0.018; 0.032	0.745	0.111	− 0.05; 0.267	0.267
SO_2_ $\left(\frac{\mu g}{{m}^{3}}\right)$	Cont^a^	0.100	− 0.07; 0.270	0.240	− 0.012	− 0.096; 0.073	0.787	− 0.007	− 0.051; 0.037	0.756	0.027	− 0.155; 0.209	0.774
	Cont^b^	0.009	− 0.04; 0.345	0.256	− 0.019	− 0.102; 0.067	0.867	− 0.011	− 0.056; 0.042	0.821	0.034	− 0.211; 0.234	0.756
	Q2^a^	0.037	− 0.056; 0.129	0.437	0.028	− 0.022; 0.077	0.271	− 0.009	− 0.035; 0.017	0.515	0.072	− 0.092; 0.236	0.393
	Q2^b^	0.042	− 0.078; 0.111	0.398	0.045	− 0.031; 0.067	0.307	− 0.015	− 0.067; 0.022	0.578	0.089	− 0.102; 0.301	0.438
	Q3^a^	− 0.015	− 0.054; 0.023	0.425	0.008	− 0.025; 0.040	0.648	− 0.013	− 0.029; 0.004	0.137	0.040	− 0.132; 0.213	0.645
	Q3^b^	− 0.021	− 0.078; 0.042	0.639	0.014	− 0.054; 0.052	0.678	− 0.015	− 0.017; 0.010	0.153	0.056	− 0.231; 0.378	0.756
	Q4^a^	− 0.05	− 0.011; − 0.040	**0.038**	0.025	− 0.009; 0.058	0.147	0.004	− 0.013; 0.021	0.630	0.005	− 0.174; 0.184	0.954
	Q4^b^	− 0.07	− 0.015; − 0.054	0.052	0.018	− 0.011; 0.078	0.129	0.006	− 0.011; 0.029	0.710	0.009	− 0.167; 0.198	0.978
NO_x_ $\left(\frac{\mu g}{{m}^{3}}\right)$	Cont^a^	− 0.047	− 0.152; 0.058	0.377	0.015	− 0.064; 0.095	0.702	0.006	− 0.036; 0.047	0.782	0.126	− 0.061; 0.312	0.176
	Cont^b^	− 0.052	− 0.167; 0.067	0.422	0.021	− 0.078; 0.112	0.811	0.008	− 0.026; 0.056	0.821	0.132	− 0.056; 0.421	0.182
	Q2^a^	− 0.055	− 0.118; 0.008	0.085	0.063	− 0.163; 0.289	0.590	− 0.087	− 0.305; 0.131	0.436	0.100	− 0.098; 0.299	0.308
	Q2^b^	− 0.045	− 0.129; 0.011	0.092	0.071	− 0.152; 0.389	0.621	− 0.092	− 0.280; 0.127	0.478	0.237	− 0.119; 0.329	0.421
	Q3^a^	0.021	− 0.020; 0.061	0.317	0.069	− 0.173; 0.311	0.570	− 0.047	− 0.309; 0.216	0.723	0.047	− 0.175; 0.269	0.673
	Q3^b^	0.023	− 0.030; 0.068	0.329	0.074	− 0.189; 0.451	0.630	− 0.076	− 0.361; 0.272	0.783	0.052	− 0.211; 0.371	0.781
	Q4^a^	− 0.012	− 0.053; 0.030	0.577	− 0.259	− 0.525; 0.007	0.053	− 0.187	− 0.421; 0.047	0.112	− 0.132	− 0.298; 0.034	0.112
	Q4^b^	− 0.018	− 0.057; 0.045	0.629	− 0.312	− 0.489; 0.011	0.058	− 0.163	− 0.539; 0.075	0.134	− 0.116	− 0.256; 0.074	0.167
CO $\left(\frac{\mu g}{{m}^{3}}\right)$	Cont^a^	0.117	− 0.017; 0.232	0.239	− 0.103	− 0.408; 0.202	0.498	0.084	− 0.152; 0.320	0.482	− 0.075	− 0.240; 0.090	0.370
	Cont^b^	0.121	− 0.012; 0.334	0.301	− 0.128	− 0.399; 0.267	0.501	0.092	− 0.178; 0.452	0.478	− 0.084	− 0.376; 0.154	0.458
	Q2^a^	0.145	− 0.064; 0.355	0.165	0.028	− 0.271; 0.326	0.851	− 0.187	− 0.419; 0.045	0.116	− 0.048	− 0.225; 0.129	0.593
	Q2^b^	0.112	− 0.051; 0.356	0.178	0.041	− 0.285; 0.421	0.784	− 0.177	− 0.359; 0.056	0.121	− 0.052	− 0.329; 0.267	0.652
	Q3^a^	− 0.106	− 0.379; 0.167	0.443	− 0.125	− 0.409; 0.159	0.381	− 0.140	− 0.369; 0.089	0.224	− 0.076	− 0.248; 0.096	0.380
	Q3^b^	− 0.112	− 0.334; 0.201	0.456	− 0.111	− 0.386; 0.145	0.356	− 0.200	− 0.452; 0.067	0.232	− 0.088	− 0.112; 0.121	0.421
	Q4^a^	− 0.049	− 0.253; 0.154	0.635	0.231	− 0.055; 0.518	0.103	− 0.085	− 0.305; 0.135	0.444	− 0.064	− 0.237; 0.109	0.461
	Q4^b^	− 0.056	− 0.348; 0.187	0.656	0.320	− 0.089; 0.694	0.219	0.112	− 0.345; 0.211	0.456	− 0.085	− 0.354; 0.209	0.529

Significant values are in bold.

^a^Model adjusted for: age, BMI, smoking, infertility diagnosis; Reference groups 1. In case Q2. Q3. Q4 reference is Q1; Q1- ≤ 25 percentile; Q2-(25–50) percentile; Q3-(50–75) percentile; Q4- > 75 percentile.

^b^Model adjusted for: age, BMI, smoking, infertility diagnosis; alcohol consumption and duration of infertility; Reference groups 1. In case Q2. Q3. Q4 reference is Q1; Q1- ≤ 25 percentile; Q2-(25–50) percentile; Q3-(50–75) percentile; Q4- > 75 percentile.

The estimated effect of PM_10_, PM_2.5_, SO_2_ exposure on antral follicle count was stronger among women whose primary infertility diagnosis was attributable to a female cause compared to women with an unexplained or male factor diagnosis (p-for-interaction 0.04, 0.02, 0.04 respectively) (Table 5). Additionally the effect of exposure to PM_2.5_ and NOx was more pronounced among women > 35 years of age compared to women < 35 years old (p-for-interaction 0.01, 0.03 respectively). PM_2.5_ affect also AMH concentration among older women (p-for-interaction 0.03) (Table 5).

**Table 5:** Effect modification of the association between exposure to air pollution and ovarian reserve parameters.

Exposure	Ozone				PM 10				PM 2.5				SO_2_				NOx				CO			
Variables	AFC	AMH	FSH	E2	AFC	AMH	FSH	E2	AFC	AMH	FSH	E2	AFC	AMH	FSH	E2	AFC	AMH	FSH	E2	AFC	AMH	FSH	E2
P for interaction																								
Age	0.23	0.41	0.62	0.21	0.07	0.85	0.93	0.48	**0.01**	**0.03**	0.83	0.76	0.83	0.56	0.39	0.33	**0.03**	0.84	0.30	0.52	0.20	0.45	0.82	0.53
BMI	0.55	0.73	0.71	0.45	0.56	0.72	0.61	0.94	0.27	0.72	0.23	0.39	0.96	0.89	0.67	0.79	0.45	0.93	0.56	0.49	0.84	0.67	0.56	0.62
Current smoking	0.48	0.34	0.36	0.45	0.39	0.43	0.62	0.39	0.51	0.76	0.84	0.43	0.26	0.62	0.29	0.39	0.93	0.72	0.39	0.58	0.86	0.39	0.10	0.81
Infertility diagnosis	0.56	0.51	0.41	0.45	**0.04**	0.81	0.81	0.74	**0.02**	0.29	0.69	0.72	**0.04**	0.39	0.45	0.49	0.29	0.48	0.29	0.25	0.78	0.21	0.30	0.67
Duration of couple’s infertility	0.31	0.42	0.49	0.45	0.98	0.78	0.53	0.83	0.31	0.85	0.39	0.42	0.56	0.20	0.73	0.86	0.30	0.10	0.59	0.50	0.20	0.67	0.26	0.51
Alcohol consumption	0.22	0.67	0.78	0.45	0.18	0.51	0.53	0.27	0.45	0.51	0.52	0.67	0.23	0.19	0.65	0.19	0.50	0.29	0.36	0.78	0.56	0.52	0.48	0.64

Significant values are in bold.

Age (< 35 years; ≥ 35 years); BMI (< 25$\frac{{\text{kg}}}{{{\text{m}}}^{2}}$, ≥ 25$\frac{{\text{kg}}}{{{\text{m}}}^{2}}$); Current smoking (Yes, No); Infertility diagnosis (Female, Male & Unexplained); Duration of couple’s infertility (1–3 years, > 3 years); Alcohol consumption (none or < 1 drink/week; 1–3 drinks/week; everyday).

## Discussion

According to our best knowledge this is one of first study which assessed ambient air pollution exposure and ovarian reserve in European population. Additionally, contrary to previous studies the ovarian reserve was examined in complex way based on different ovarian reserve parameters (AFC, AMH, FSH, and E2). Also different air pollutants levels were evaluated (ozone, PM_10_, PM_2.5_, SO_2_, NO_x_, CO). Negative association between exposure to PM_2.5_ and AMH levels and AFC were found. Additionally, exposure to SO_2_ in the 4th quartile of exposure compared to the first one decrease the AFC. The results also suggest that PM_10_, PM_2.5_, SO_2_ exposure on antral follicle count may be more pronounced among women with a female factor infertility diagnosis. Additionally, exposure to PM_2.5_ and NOx on AFC and AMH was stronger among older women (> 35 years of age).

Four epidemiological studies were performed to assess the air pollution exposure and ovarian reserve^24–27^. The first study was performed by Gaskins et al.^24^, the author observed significant negative relationship between exposure to PM_2,5_ and antral follicle count. The association of PM_2.5_ with antral follicle count was stronger among women with female factor infertility and abnormal menstrual cycles. The observed results are similar to our study regarding to PM_2.5_ exposure and more pronounced effect among female with female factor of infertility. Abareshi et al.^25^ observed inverse relationship between exposure to air pollution (PM_1_, PM_2,5_) and levels of Anti-Müllerian hormone (AMH). In the study, the author examined only particulate matters exposure (PM_1_, PM_2,5_ and PM_10_) in population of 67 women^25^. Another study performed in Korea found that exposure to PM_10_ and PM_2,5_ decrease ovarian reserve assessed only by AMH concentrations^26^ which is in line with our results. In the study by Feng et al.^27^ only the antral follicular count (AFC) and air pollution exposure were evaluated. In the study SO_2_ exposure was associated with lower antral follicle count^27^. Similar relationship was observed in our study however the duration of exposure was different than in our study. The study covered of long-term exposure to ambient air (from 2013 to 2019), whereas in our study for each pollutant, the average value for the 6 months which address the period of developmental stage from primary follicle to antral follicle was assessed. The study by Feng et al.^27^ evaluated only AFC, as the main parameter of ovarian reserve.

The study performed in animals demonstrated that exposure to PM may reduce the antral follicle count in mice^29^. Another study in mice found diminishing ovarian reserve^32^ after exposure to diesel exhaust. Furthermore, Zhou et al.^33^ observed ovarian dysfunction associated with chronic PM_2,5_ exposure (4 months). The causal relationship that links air pollution to ovarian reserve has yet to be elucidated. Prior reports have suggested that folliculogenesis can be impaired by the increased oxidative stress and cellular apoptosis induced by ambient air containing a range of pollutants^34^. ROS can damage the oocytes and can lead to negative impact on female reproduction^35^. In results oxidative stress (OS) can lead telomere dysfunction (DNA damage or aberrant telomere recombination) and caused miscarriages and infertility^36^. Basically, there are three ways how the environmental pollutants can impact ovarian function: by causing endocrine disrupting effect, by induction of oxidative stress and by causing epigenetic modification^37^. Thus according to study performed by Styszko et al.^38^, PM_10_ and PM_2,5_ fractions of aerosols shows high oxidative potential (OP) and also included metals in composition such as: K, Ca, Ti, V, Mn, Fe, Pb, Cu, Cr, Zn, Hg, Ni, some of these metals can as endocrine disrupting chemicals so via the impact on the hormone level the ovarian reserve can be diminished^39^. Hence, endocrine disruptors can alter hormone receptor binding and action, such as: mainly on the aryl hydrocarbon receptor (AHR), estrogen receptors (ERs) or androgen receptor (AR), which can lead directly on disturb ovarian functions^40^. The studies in mice suggest that aryl hydrocarbon receptor (AHR) can play a function in the formation primordial follicles and also regulation of antral follicle count^41^. Other study reported that the AHR can regulate follicle growth through change estradiol biosynthesis pathway factors. The study performed by Revelli et al., suggest that estrogens and progesterone lead in the intraovarian regulation of follicle growth^42^. According to study Walters et al., the androgen receptor can lead to follicular growth and follicle and ovulation development^43^.

In present study the ovarian reserve was assessed by specialist in gynaecological endocrinology and reproduction, which result in the reliable assessment of antral follicle count. Moreover this is the first study which assess all parameters of ovarian reserve such as: Anti-Müllerian Hormone (AMH), follicle-stimulating hormone (FSH) and antral follicle count (AFC). AFC has been well-established biomarker for reflecting ovarian reserve from the aspect of visualization^44^. Also AMH is a reliable predictor for reflecting ovarian reserve due to its invariability with cyclical menstrual cycles^45^ and is associated with follicular size^46^.

The levels of air pollution which were found in present study (Ozone mean: 44,8 µg/m^3^, PM_10_ mean: 38,98 µg/m^3^, PM_2,5_ mean: 31,22 µg/m^3^, SO_2_ mean: 41,56 µg/m^3^, NO_x_ mean: 35,70 µg/m^3^, CO mean: 0,48 µg/m^3^) are comparable with Abareshi et al., the median IQR of PM_2,5_ (42,2 µg/m^3^) and PM_10_ (47,4 µg/m^3^) concentrations^25^. Kim et al. assessed PM_10_, PM_2,5_, SO_2_, CO and ozone, but only two pollutants were in similar range PM_10_ (46,7 µg/m^3^) and ozone (37,5 µg/m^3^), the level of PM_2,5_ (25,8 µg/m^3^) and SO_2_ (4,4 µg/m^3^) were lower, and one higher CO (62,1 µg/m^3^)^26^. In the study carried out by Gaskins et al. the level of PM_2,5_ was lower than in present study (9 µg/m^3^)^24^. In the China study only SO_2_ were similar (53 µg/m^3^) compared to our study. In case of PM_10_, PM_2,5_, CO and ozone the levels were higher (108 µg/m^3^, 61 µg/m^3^, 120 µg/m^3^, 120 µg/m^3^)^27^.

The study has several strengths such as: performing the study in one fertility center, biological samples were collected and analyzed using the same procedure. The qualified medical specialist (infertility specialist) was assessed ovarian reserve according to the same standardized protocol. All procedures were carried out at the 2–4 days of the cycle (beginning of follicular phase). Moreover all participants answered the same questionnaire about sociodemographic, lifestyle and medical risk factors thus, we allowed for controlling potential confounding factors in statistical model.

The study has also some limitations. First, the study was performed in fertility clinic, which may not lead to generalized the results directly to general population. Second,, air pollution was assessed based on the information about ZIP code of residence for each women and was linked to the nearest air monitoring station. This method may not constitute the actual levels of exposure because we are not staying all the time in our places of residents. More proper measurement would minimize such a potential measurement bias. Nevertheless this is the popular method used in epidemiological studies with big sample size, where the individual monitoring may be not comfortable for participants and expensive^24–27,47–49^.

## Conclusions

In summary, we observed that the exposure to PM_2,5_ decrease antral follicle count and AMH concentration, whereas the SO_2_ exposure was also negatively related only to antral follicle count. The results rise of importance to reduce the potential risk of ambient air pollution exposure and suggest the need for more strict regulations and controls forced by appropriate law. Additionally, further studies involving general population and underlying the mechanism of adverse impact on ovarian reserve are needed.

## Data Availability

The datasets used and analyzed during the current study are available from the corresponding author upon reasonable.
