# Author Correction: Exposure to air pollution and ovarian reserve parameters

**DOI:** 10.1038/s41598-024-53558-3

**Published:** 2024-02-12

**Authors:** Katarzyna Wieczorek, Dorota Szczęsna, Michał Radwan, Paweł Radwan, Kinga Polańska, Anna Kilanowicz, Joanna Jurewicz

**Affiliations:** 1 Department of Chemical Safety, Nofer Institute of Occupational Medicine, 8 Teresy St, 91‑348 Łódź, Poland; 2 Department of Gynecology and Reproduction, “Gameta” Hospital, 34/36 Rudzka St, 95‑030 Rzgów, Poland; 3 Faculty of Health Sciences, Mazovian State University in Płock, 2 Dabrowskiego Sq, 09‑402 Plock, Poland; 4 Department of Gynecology and Reproduction, Gameta” Health Centre, 7 Cybernetyki St, 02‑677 Warsaw, Poland; 5 Department of Gynecology and Reproduction, “Gameta” Clinic, Kielce-Regional Science–Technology Centre, 45 Podzamcze St, 26‑060 Chęciny, Poland; 6 Department of Paediatrics and Allergy, Copernicus Memorial Hospital, Medical University of Lodz, Piłsudskiego 71, 90‑329 Lodz, Poland; 7 Department of Toxicology, Medical University of Lodz, Muszynskiego 1, 90‑151 Łódź, Poland

Correction to: *Scientific Reports* 10.1038/s41598-023-50753-6, Published online 03 January 2024

In the original version of this Article, the Anti-Müllerian hormone ‘(AMH)’ was misspelt as ‘(AHM)’.

As a result, the Abstract

“Significant negative association between PM_2,5_ and AHM (p = 0.032), as well as AFC (p = 0.044), was observed. Moreover, SO_2_ concentrations decrease AFC (p = 0.038).”

now reads,

“Significant negative association between PM_2,5_ and AMH (p = 0.032), as well as AFC (p = 0.044) was observed. Moreover, SO_2_ concentrations decrease AFC (p = 0.038).”

In addition, the Introduction

“The studies on mice suggest that exposure to fraction PM_2,5_ can reduce the level of Anti-Müllerian hormone (AHM)^28^.”

now reads,

“The studies on mice suggest that exposure to fraction PM_2,5_ can reduce the level of Anti-Müllerian hormone (AMH)^28^.”

The original Article has been corrected.

